# KSHV PAN RNA Associates with Demethylases UTX and JMJD3 to Activate Lytic Replication through a Physical Interaction with the Virus Genome

**DOI:** 10.1371/journal.ppat.1002680

**Published:** 2012-05-10

**Authors:** Cyprian C. Rossetto, Gregory Pari

**Affiliations:** The Department of Microbiology & Immunology, University of Nevada, Reno School of Medicine, Reno, Nevada, United States of America; University of North Carolina, United States of America

## Abstract

Kaposi's sarcoma-associated herpesvirus (KSHV) is the cause of Kaposi's sarcoma and body cavity lymphomas. KSHV lytic infection produces PAN RNA, a highly abundant noncoding polyadenylated transcript that is retained in the nucleus. We recently demonstrated that PAN RNA interacts with several viral and cellular factors and can disregulate the expression of genes that modulate immune response. In an effort to define the role of PAN RNA in the context of the virus genome we generated a recombinant BACmid that deleted the PAN RNA locus. Because of the apparent duplication of the PAN RNA locus in BAC36, we generated BAC36CR, a recombinant BACmid that removes the duplicated region. BAC36CR was used as a template to delete most of the PAN RNA locus to generate BAC36CRΔPAN. BAC36CRΔPAN failed to produce supernatant virus and displayed a general decrease in mRNA accumulation of representative immediate early, early and late genes. Most strikingly, K-Rta expression was decreased in lytically induced BAC36CRΔPAN-containing cell lines at early and late time points post induction. Expression of PAN RNA in *trans* in BAC36CRΔPAN containing cells resulted in an increase in K-Rta expression, however K-Rta over expression failed to rescue BAC36CRΔPAN, suggesting that PAN RNA plays a wider role in virus replication. To investigate the role of PAN RNA in the activation of K-Rta expression, we demonstrate that PAN RNA physically interacts with the ORF50 promoter. RNA chromatin immunoprecipitation assays show that PAN RNA interacts with demethylases JMJD3 and UTX, and the histone methyltransferase MLL2. Consistent with the interaction with demethylases, expression of PAN RNA results in a decrease of the repressive H3K27me3 mark at the ORF50 promoter. These data support a model where PAN RNA is a multifunctional regulatory transcript that controls KSHV gene expression by mediating the modification of chromatin by targeting the KSHV repressed genome.

## Author Summary

Kaposi's sarcoma associated herpesvirus (KSHV) is the cause of Kaposi's sarcoma and body cavity lymphoma. KSHV lytic infection produces polyadenylated nuclear RNA (PAN RNA), a highly abundant long noncoding RNA that was recently shown to interact with several viral and cellular proteins. Expression of PAN RNA in cell lines affects gene expression of several factors that associated with immune response. In this study we have now generated a recombinant BACmid clone that has the PAN RNA locus deleted. This recombinant BACmid (BAC36CRΔPAN) failed to efficiently reactivate and was deficient for the production of progeny virus and expressed a low level of K-Rta mRNA when compared to wild-type KSHV BACmid. The overexpression of K-Rta in BAC36CRΔPAN containing cell lines failed to complement the null phenotype. We used the method “Chromatin Isolation by RNA purification” (ChIRP) we show that PAN RNA physically interacts with the ORF50 promoter. RNA chromatin immunoprecipitation assays show that PAN RNA interacts with the demethylases UTX and JMJD3 and the histone methyltransferase MLL2. Data strongly suggests that PAN RNA acts to transcriptionally activate KSHV gene expression by acting as a molecular scaffold for chromatin modifying enzymes and relieve suppression by removing the H3K27me3 mark.

## Introduction

Kaposi's sarcoma associated herpesvirus (KSHV) is an oncogenic herpesvirus that is the cause of Kaposi's sarcoma and body cavity lymphoma [1]–[4]. KSHV is primarily latent in cell culture but can be induced to undergo lytic DNA replication by treatment of cells with a variety of chemical agents or by the expression of the lytic switch protein encoded by ORF50, K-Rta [5]. Lytic replication results in the expression of genes that are involved in DNA synthesis, virus maturation and the production of infectious virus [6]. Upon lytic induction, KSHV expresses a very abundant nuclear long noncoding RNA (lncRNA) referred to as polyadenylated nuclear RNA or PAN RNA (Nut-1 or T1.1) [7], [8]. The expression of PAN RNA appears to be regulated by K-Rta through a cis acting element present within the promoter region for the transcript and by ORF57, which stabilizes PAN RNA [8]–[14]. PAN RNA accumulates to very high levels in the presence of ORF57 and was recently shown to interact with several viral and cellular factors and to act as a regulatory transcript, mediating changes in expression of genes involved in immune modulation [12], [15]. Additionally, PAN RNA was demonstrated to interact with poly-A binding protein C1 PABPC1 [12], [16]. The KSHV encoded shutoff exonuclease protein (SOX) was shown to upregulate PAN RNA expression in transient assays, although PAN RNA itself does not contribute to host shutoff [16]. Knockdown of PAN RNA in virus-infected cells using antisense oligonucleotides apparently results in a decrease in both virus late RNA accumulation and infectious virion production [16]. This result suggests that PAN RNA plays in important role in virus replication, however full evaluation of PAN RNA and its contribution to KSHV growth can only be achieved by generation of a recombinant virus knockout of the PAN RNA locus.

Although many viral factors contribute to virus growth, the evaluation of the contribution of specific virus encoded factors toward virus replication is traditionally performed by generating a recombinant virus or BACmid that mutates or deletes the gene of interest. For herpesviruses, including KSHV, the generation and manipulation of an infectious BACmid clone is invaluable to genetic characterization of virus gene expression. Our laboratory and others have successfully used the KSHV BACmid BAC36 to evaluate the role of cis and trans acting factors in virus growth [17]–[19]. Recently it was discovered that BAC36 contains a duplicated genomic region that includes part of ORF19, ORFs 18, 17, 16, K7, K6, and K5 [20]. This BAC36 duplication also includes the locus that encodes PAN RNA, which partially overlaps with the K7 ORF [20]. Hence the mutation or deletion of these ORFs or the PAN RNA locus within the BAC36 genome is complicated by this duplication.

In this report we have now used the BAC36 template to generate a new recombinant BACmid, BAC36CR, which has the duplicated region removed and leaves only the BACori and the cassette that encodes the chloramphenicol resistance gene, hygromycin resistance gene and the green fluorescent protein ORF (GFP). BAC36CR establishes latency and cell lines can be selected that upon reactivation express lytic genes and produce infectious virus. BAC36CR was used to generate a recombinant virus that has most of the PAN RNA coding region removed. This PAN RNA knockout virus, BAC36CRΔPAN failed to produce infectious virus and showed decreased expression levels of K-Rta as well as early and late transcripts. Transient transfection of PAN RNA in BAC36CRΔPAN cell lines transactivated the ORF50 promoter, however expression of K-Rta in *trans* failed to complement BAC36CRΔPAN, suggesting a wider role for PAN RNA in virus replication. Using a method to identify the binding substrates for RNA, “Chromatin Isolation by RNA Purification” (ChIRP), we show that PAN RNA physically interacts with the ORF50 promoter. We also demonstrate that PAN RNA interacts with demethylases UTX and JMJD3 and the histone methytransferase MLL2. These data strongly suggest that PAN RNA activates KSHV gene expression by mediating the removal of the repressive H3K27me3 mark while simultaneously marking H3K4me3, a mark associated with gene activation.

## Materials and Methods

### Cells and plasmids

293L cells containing BACmid constructs were maintained in Dulbecco's modified Eagle medium supplemented with 10% fetal bovine serum and 125 ug/ml hygromycin B (PhytoTechnology Laboratories). BAC36, the wild type HHV8 BACmid, was provided by S. Gao (University of Southern California). TREx/BCBL-1 Rta cells, provided by J. Jung (University of Southern California) were maintained in RPMI supplemented with 10% FBS and 20 ug/ml hygromycin B. Where noted, 293L BAC containing cells were reactivated with 25 ng/ml TPA (Phorbol 12-myristate 13-acetate) and 0.3 mM n-butyrate (Sigma Aldrich).

### Removal of duplicated region within BAC36

To remove the duplicated region within BAC36, primers were designed with 40–50 nucleotides of homology to the target locus while allowing for the amplification of the GalK-KanR cassette by including 20-nt of homology to GalK-KanR plasmid. Primers used to target removal of the duplicated region (Bold uppercase is homologous to duplicated region, lowercase is homologous to GalK-KanR plasmid) forward primer: **ATAAAACGCAGCTCTTGGGCGTGGAATGTATTTAAATTCTTATTTTGCAAAAAG**cctgttgacaattaatcatc, reverse primer: **GATTCACATTGTTTAGAGAGAGTTTTTCTTAGTCACCATTCCATACTTGGGCAGTATT**ctcagcaaaagttcgattta. After amplifying the GalK-KanR cassette using Easy-A taq polymerase (Stratagene), the PCR product was subjected to DpnI cleavage and subsequent agarose gel purification to remove all residual plasmid template. The GalK-KanR cassette was used to electroporate into the competent EL350 cells.

EL350 bacteria containing BAC36 were made competent by growing a 1∶250 dilution of an overnight culture in SOC media with 30 µg/ml chloramphenicol shaking at 30°C until it reached an O.D. equal to 0.6. The BAC containing bacteria were then placed in a 42°C water bath for 15 minutes to allow for the induced expression of the temperature sensitive recombination enzymes. After 15 minutes the bacteria were placed in an iced-water slurry for 5 minutes to completely cool the culture. The bacteria was then pelleted at 2500 rpm for 10 minutes in a table top centrifuge that had been pre-chilled to 0°C, the media was removed and the cells were gently resuspended in 10% glycerol. This was repeated for a total of four washes, after the last wash the tube was inverted and only the residual liquid was used to resuspend the bacteria (∼50 µl). The PCR product was transformed into the bacteria using a Bactozapper Cloning Gun (Tritech Research), cells were recovered in LB for 1 hour at 30°C in a shaking incubator. After 1 hour cells were pelleted and resuspended in residual liquid before plating onto kanamycin (50 µg/ml) and chloramphenicol (30 µg/ml) LB-agar plates and grown overnight at 30°C. Colonies that grew were picked and grown for in LB for further analysis. 10–12 ml of overnight culture were pelleted and DNA was isolated using alkaline lysis of bacteria and phenol-chloroform extraction to purify the DNA. The DNA was subjected to restriction enzyme cleavage and separated on a 0.8% agarose gel. The ethidium bromide stained DNA agarose gel was UV-visualized to assess the DNA for any large deletions and overall quality of DNA. The DNA was transferred to a nylon membrane and hybridized with ^32^P dCTP-labeled probes, either a GalK probe or PAN probe. Membranes were incubated overnight at 65°C in hybridization buffer, washed and imaged using a Storm Scanner Phosphoimager (GE).

Once the DNA was confirmed to have the correct insert of GalK-KanR within the duplicated region of BAC36, a large-scale purification (Qiagen Large Construct Kit) was used to obtain quality DNA to electroporate into SW106 cells. The SW106 cells, which have a deleted galactokinase gene, are necessary for the negative selection of any recombinants that still harbor the GalK-KanR cassette. Once it was confirmed that the BAC DNA was successfully harbored in the SW106 cells, they were again made competent and induced to express the recombination enzyme as state above, but this time a double stranded DNA duplex (IDT) was used to replace the GalK-KanR cassette (bold uppercase letters denote regions homologous to the hygromycin sequence end within the BAC and lowercase letters are denote regions homologous to the duplicated region towards the terminal repeat): **ACCCCCAAAAACCACCGCGCGGATTTCTGGCGCCAGTGCCAAGCTG**tgtgcttgtgactgatacaaatcgccatacatggcaacaggttg. After electroporation with 200 ng of the duplex, bacteria cells were recovered for four hours in LB, shaking at 30°C. To remove any remaining carbon source that the bacteria could alternatively use the bacterial cells were pelleted and wash four times with 1X M9 salt solution. After the final wash the cells were resuspended in residual liquid and plated onto counter selection plates (1X M63, 15 g/L agar, 0.2% 2-deoxy-galactose (DOG), 0.2% glycerol, 1 mg/L D-Biotin, 45 mg/L L-Leucine, 1 mM MgSO_4_^.^7H_2_O, and 30 ug/ml chloramphenicol). After 4 days, colonies were picked, BAC minipreps were performed, and correct BAC recombinants were confirmed by southern blot analysis and sequencing.

### Generation of PAN deletion BACmid

Generation of the PAN deletion BACmid is essentially the same as described above except as noted. To remove the majority of the PAN RNA locus region within BAC36CR, primers were designed with 40–50 nucleotides of homology to the target locus while allowing for the amplification of the GalK-KanR cassette by including 20-nt of homology to GalK-KanR plasmid. Primers used to target removal of PAN forward primer (bold uppercase is homologous to the PAN locus, lower case is homologous to the GalK-KanR plasmid): **GCATTGGATTCAATCTCCAGGCCAGTTGTAGCCCCCTTTTATGATATGCG**aataaacctgttgacaattaatcatc, reverse primer: **TGCATTACGTTATGGTATTAGTTTAATTTGAGCTCTAGGCACGTTAAATT**ctcagcaaaagttcgattta. The primers used removes a 634 nt internal region of PAN, spanning nt 29022–29656 (GenBank accession no. AF148805). The GalK-KanR cassette targeting the PAN locus was electroporated into competent SW106 that contained BAC36CR. PAN deletion mutants were first selected on chloramphenicol/kanamycin agar plates; correct insertional mutants were confirmed with southern blot analysis and subsequent sequencing confirmation. To complete the process the GalK-KanR cassette was replaced with a double stranded DNA duplex (IDT): GATTCAATCTCCAGGCCAGTTGTAGCCCCCTTTTATGATATGCGAATTTAACGTGCCTAGAGCTCAAATTAAACTAATACCATAACGTAA. In addition a PAN revertant was created by using a PAN PCR product that re-introduced the complete PAN locus where the GalK-KanR was inserted. These BAC recombinants were selected on counter selection plates, and correct colonies were picked and subjected to confirmation by southern blot analysis and sequencing.

### Southern blot analysis

PstI or BamHI cleaved BAC DNA was separated on 0.8% agarose gel using 1X TAE buffer and visualized by ethidium bromide staining. The gel was denatured and transferred to Zeta-Probe GT blotting membrane (Bio-Rad). DNA probes were radiolabeled with [α-^32^P]dCTP (Perkin Elmer) with Rediprime II random prime labeling system (GE Healthcare), either a GalK probe (Forward primer: CTTAACGGTCAGGAAGCAG and Reverse primer: CAGCACTGTCCTGCTCCTTG) or PAN probe (Forward primer: CGTGGCAAGCAGTACGCTAACGCA and Reverse primer: AAAGCCCACAAAGGCGCCACAGCG). Hybridization of the probe to the membrane was performed rotating overnight at 65°C in hybridization buffer (10% polyethylene glycol, 7% sodium dodecyl sulfate [SDS], 1.5× SSPE). DNA blots were hybridized with either the GalK or PAN probes. The blots were washed twice for 15 minutes with 2×SSC-0.1% SDS and twice for 30 minutes with 0.1×SSC-0.1%SDS at 65°C. The blots were imaged using a phosphoimager (GE Healthcare).

### Real-time PCR

2×10^6^ 293L BAC containing cells were plated in a 10-cm tissue culture dish and induced with the use of 0.3 mM Sodium Butyrate. For the experiments where K-Rta expression plasmid was added, 5 µg of pXi-ORF50-Flag was transfected into 293L BAC containing cells using TransIT-LT1 (mirus) according to manufactures instructions. Total RNA was harvested from 293L BAC containing cells using PureLink RNA mini kit (Invitrogen), followed by removed of genomic DNA using Turbo DNA-free (Ambion). cDNA was synthesized from 5 µg of total RNA in the presence of random hexamers, dNTPs, and Superscript III reverse transcriptase (Invitrogen). The resulting cDNA was then used along with TaqMan Universal PCR Master Mix (Applied Biosystems) and specific primers and FAM labeled probes (IDT) in an Eppendorf RealPlex. The following qPCR program was used: one cycle 95°C hot start for 5 minutes, and forty cycles of 95°C for 15 seconds and 60°C for 1 min. Primers and probes used for qPCR detection of specific gene expression are shown in Table 1.

**Table 1:** qPCR primers and probes used to evaluate BACmid gene expression.

Gene	Forward Primer	Reverse Primer	Probe
ORF50	ACC AAG GTG TGC CGT GTA GAG ATT	AGC CTT ACG CTT CTT TGA GCT CCT	/56-FAM/AGG CGA CAA /ZEN/CAC CCA AAC GAA AGC A/3IABkFQ/
LANA	AAC AAA TTG CCA GTA GCC CAC CAG	TAA CTG GAA CGC GCC TCA TAC GAA	/56-FAM/ATA CAC CAG /ZEN/ACG ATG ACC CAC AAC CT/3IABkFQ/
PAN	ATA GGC GAC AAA GTG AGG TGG CAT	TAA CAT TGA AAG AGC GCT CCC AGC	/56-FAM/ACA TTG GAC /ZEN/TAA AGT GGT GTG CGG CA/3IABkFQ/
K7	GCC AGC TTG AGT CAG TTT AGC ACT	AAG CGA CGC AAT CAA CCC ACA ATC	/56-FAM/TTG GCT GCC /ZEN/GCT TCA CCT ATG GAT TT/3IABkFQ/
K8	ATC AGT CAC ATT CTC CCA CGC GAA	TAC CTG CTG CAG CTG TCT TGT GTA	/56-FAM/ATA CGG CCG /ZEN/CGT GTC ATC GAA AGC A/3IABkFQ/
ORF25	GCT TGC AAT AAG CAC CAC ATC GGT	AAC TGC GAG AAC CGT GTC CAC TAA	/56-FAM/ACG TCG GCT /ZEN/TTG CAG TTT GGT ATG GA/3IABkFQ/
ORF57	AGA GGC ATG TAA CCT TCT TGG CGA	ATA CTT CTG CGA CTC TGC ATG CCT	/56-FAM/GCT GCT CTT /ZEN/GGC CTT TGT CCT AAC TA/3IABkFQ/
ORF9	AAG GGC GAA AGA TGC TGG AGA GAT	AGT CTG ACA CGC TGT CCA TGT TGA	/56-FAM/AGG TCA TAT /ZEN/ACG GCG ACA CTG ACT CT/3IABkFQ/
ORF37	TGA CAC CCT GGA TGG GTT AAC AGT	TCT CGA ACC TTG GCG TGC TTT AGA	/56-FAM/AGG CTG TCC /ZEN/TCG CAA GCT TGA GCT TT/3IABkFQ/

### Protein analysis

5×10^6^ cells containing BAC36CR or BAC36CRΔPAN were treated with 0.3 mM sodium butyrate to induce lytic reactivation. Three days post induction cells were lysed in RIPA buffer (50 mM Tris-HCl pH 8.0, 150 mM NaCl, 2 mM EDTA, 1% NP-40, 0.5% sodium deoxycholate, 0.1% SDS) with Protease Inhibitor Cocktail (Sigma), one volume of lysate was mixed with one volume of Laemmli buffer with 5% β-mercaptoethanol and subsequently boiled for 5 minutes before loading onto a 10% SDS-PAGE gel. Samples were transferred to a PVDF membrane and probed with antibodies (K-Rta, K-ZIP, LANA, and actin). Secondary antibodies used for either chemiluminescence or fluorescence imaging (Li-Cor Odyssey).

### Viral DNA accumulation

3×10^6^ BAC containing 293L cells were plated into 10-cm tissue culture dish. 24 hrs after plating cells were transfected with 5 µg of ORF50 mammalian expression plasmid (pXi ORF50-Flag) and 5 µg of PAN mammalian expression plamid (pcDNA-PAN) using TransIT-LT1 (Mirus) according to manufactures' instructions. 4 days post transfection virus was harvested from the media, first a low speed tabletop centrifugation (1000× g for 10 minutes) was done twice remove any cells. Then the supernatant was centrifuges for 90 minutes in an SW41Ti rotor at 15,000 RPM. After centrifuging the media was removed and the virus pellet was resuspended in TE and subjected to DNase treatment (Turbo DNA, Ambion) to remove contaminating DNA, and then viral DNA was extracted from the purified virus pellet. One equal volume of DNA cell extraction buffer (2% SDS, 10 mM EDTA, and Tris-HCl [pH 8.0]) was proteinase K (150 ug/ml) was added to the virus suspension and incubated at 65°C for 1 hr. DNA was extracted using phenol∶choloform∶isoamyl alcohol (24∶24∶1) followed by ethanol precipitation at −20°C for 15 minutes, and spun down 10 minutes 13,000 RPM in a micro-centrifuge. The resulting viral DNA pellet was resuspended with 10 ul of TE and used for qPCR analysis using the ORF25 and LANA TaqMan primers and probes. BAC36CR DNA was used to create a standard curve.

### Chromatin Immunoprecipitation (ChIP)

5×10^6^ cells containing BAC36CR or BAC36CRΔPAN were treated with 0.3 mM sodium butyrate to induce lytic reactivation. Three days post induction cells were harvested and washed once with 1× PBS, and fixed in 1% methanol-free formaldehyde for 10 minutes. Cells were pelleted, washed once with PBS and quenched with 125 mM glycine for 5 minutes. After a final wash with PBS cells were brought up in 2 mL RIPA buffer (50 mM Tris-HCl pH 8.0, 150 mM NaCl, 2 mM EDTA, 1% NP-40, 0.5% sodium deoxycholate, 0.1% SDS) with Protease Inhibitor Cocktail (Sigma), and 1 mM phenylmethanesulphonylfluoride (PMSF) and subjected to sonication to produce chromatin in the size range of 400–600 bp. Chromatin was precleared with mouse IgG-AC (Santa Cruz Biotechnology) at 4°C for 30 min. For each immunoprecipitation, approximately 2 µg of antibody (anti-JMJD3 [AbCam], anti-UTX [AbCam], and anti-K-Rta) was incubated with the lysate at 4°C overnight. An antibody isotype control immunoprecipitation was also performed. Magnetic protein-G beads (Active Motif) were blocked with 100 µg/ml sheared salmon sperm DNA and 1 mg/ml BSA at 4°C overnight then washed with RIPA buffer. The blocked and washed protein-G beads were incubated with the lysate rotating at room temperature for 1 h. The beads were washed once with low salt buffer (0.1% SDS, 0.1% Triton-X 100, 2 mM EDTA, 20 mM Tris pH 8, 150 mM NaCl), once with high salt buffer (0.1% SDS, 0.1% Triton-X 100, 2 mM EDTA, 20 mM Tris pH 8, 500 mM NaCl), once with LiCl buffer (0.25 M LiCl, 1% NP-40, 1% deoxycholate, 1 mM EDTA, 10 mM Tris pH 8) and twice with TE. Beads were resuspended in TE, incubated with 5 µg RNase A (Affymetrix) at 37°C for 30 minutes, then incubated with 50 µg proteinase K (US Biologicals) and 10% SDS at 65°C overnight to reverse crosslink. For input control samples, NaCl was added to the sonicated lysate to a final concentration of 0.3 M, and incubated at 65°C overnight. DNA was extracted with equal volume phenol∶chloroform∶isoamyl (Affymetrix) and precipitated with 100% ethanol. Eluted DNA was resuspended in 50 µl of TE and used for PCR analysis. The following PCR primers were use, ORF50 promoter (Forward: GGTACCGAATGCCACAATCTGTGCCCT, Reverse: TTTGTGGCTGCCTGGACAGTATTC), and ORF45 (Forward: ACGTCCGGAGAGTTGGAACTGTCA, Reverse: GGGGTCCATGGGATGGGTTAGTCA).

### DNA ChIP for H3K27me3 analysis

For the H3K27me3 ChIP, 5×10^6^ BAC36CR or BAC36CRΔPAN containing 293L cells were transfected with either K-Rta expression plasmid pXi-ORF50-flag and/or PAN expression plasmid pcDNA-PAN (provided by N. Conrad, University of Texas). Three days post transfection, cells were treated as described above, except ChIP-grade H3K27me3 (Abcam) specific antibody was used for the immunoprecipitation. Analysis of fold enrichment was calculated by normalizing each sample to it's input DNA and relative amounts of enriched H3K27me3 where compared to the value of H3K27me3 from untransfected (untreated) BAC36CR.

### RNA Cross-Linking Immunoprecipitation (RNA CLIP)

1×10^7^ TREx/BCBL-1 Rta cells were treated with 1 µg/ml Doxycyline (Clonetech) to induce expression of K-Rta and subsequent lytic reactivation. Three days post induction cells were harvested and washed once with 1× PBS, and fixed in 1% methanol-free formaldehyde for 10 minutes. Cells were pelleted, washed once with PBS and quenched with 125 mM glycine for 5 minutes. After a final wash with PBS cells were brought up in 2 mL RIPA buffer (50 mM Tris-HCl pH 8.0, 150 mM NaCl, 2 mM EDTA, 1% NP-40, 0.5% sodium deoxycholate, 0.1% SDS) with Protease Inhibitor Cocktail (Sigma), RNase Out (Invitrogen) and 1 mM phenylmethanesulphonylfluoride (PMSF). Cells were sonicated and the extract was centrifuged at 800× g for 5 minutes at 4°C to remove debris. RNA-protein complexes were precipitated by adding 300 µl lysate, 5 µl antibody, 25 µl Protein G magnetic beads (Active Motif), and 1 µl RNase Out. This mixture was rotated 1 hr at 4°C. Input control was 98 µl of lysate mixed with 2 µl of 5 M NaCl, and frozen at −80°C until proteinase-K digestion step. After incubation the beads were washed with 1 mL of RIPA buffer, once with 1 mL of low salt wash (0.1 SDS, 1% TritonX100, 2 mM EDTA, 20 mM Tris [pH 8.0], 150 mM NaCl), once with 1 ml of high salt wash (0.1 SDS, 1% TritonX100, 2 mM EDTA, 20 mM Tris (pH 8.0), 500 mM NaCl), once with 1 ml of LiCl wash (0.25 m LiCl, 1% NP40, 1% sodium deoxycholate, 1 mM EDTA, 10 mM Tris [pH 8.0]) and twice with 1 mL of TE. The beads were resuspended in 150 µl elution buffer (1% SDS, 100 mM NaHCO_3_ pH 9.0) for 15 minutes. The elution step was repeated and the fractions combined, 60 µl 1 M Tris-HCl (pH 6.8) was added to the elution complexes, proteinase-K was added to 0.2 µg/ml to the samples and input, and the samples were incubated at 37°C for 60 minutes. Crosslinking was reversed at 65°C for 18 hours. The beads were then pelleted and supernatant was moved into 1 ml Trizol LS (Invitrogen) and incubated for 5 minutes at room temperature. 250 µl of chloroform was added to the Trizol mixture, rotated by hand and allowed to incubate for 15 minutes before centrifuging 12,000× g for 10 minutes at 4°C to separate the phases. The upper phase containing the RNA was removed and 1× volume of isopropanol was used to precipitate the RNA, 1 µl of GlycoBlue (Ambion) was added to aid in visualizing the RNA pellet. After 15 minutes incubation at room temperature samples were centrifuged at 12,000× g for 15 minutes at 4°C, and then wash with ice-cold 75% ethanol. The pellet was briefly allowed to air-dry and then resuspended in 30 µl nuclease-free water. RNA samples were treated with Turbo DNA-Free DNase (Ambion) according to manufacturers instructions. After quantification of RNA using a Nano-drop (Thermo Scientific), 5 µl of the RNA was then used in Qiagen OneStep RT-PCR Kit, using primers to an internal region within the PAN locus (Forward: TAA TGT GAA AGG AAA GCA GCG CCC, Reverse: TAACATTGAAAGAGCGCTCCCAGC), ORF45 (Forward: ACGTCCGGAGAGTTGGAACTGTCA, Reverse: GGGGTCCATGGGATGGGTTAGTCA), and U1 (Forward: ATACTTACCTGGCAGGGGAG, Reverse: CAGGGGAAAGCGCGAACGCA). The no-RT control was subjected only to PCR and not the reverse transcriptase step.

### Chromatin Isolation by RNA Purification (ChIRP)

The ChIRP protocol was adapted for PAN RNA hybridization and pull down from the original published manuscript by Chu et al. [21]. All probes were biotinylated at the 3′ end using an 18-carbon spacer arm. Probes were designed against PAN full-length RNA (antisense) sequence using the online designer at http://www.singlemoleculefish.com and synthesized at the Protein and Nucleic Acid Facility (Stanford University). Table 2 shows the oligonucleotide hybridization probes. Briefly, 20×10^6^ TREx/BCBL1 Rta cells were treated with 1 µg/ml Doxycycline to induce the expression of K-Rta and subsequent lytic reactivate of KSHV virus. Three days post induction cells were crosslinked with 1% glutaraldehyde in PBS for 10 min at room temperature. The glutaraldehyde was removed and the crosslinking was quenched with 125 mM glycine in PBS for 5 minutes at room temperature. Cells were washed twice with ice-cold PBS and pelleted at 2500× g, cell pellets were snap frozen in liquid nitrogen and stored at −80°C. The cell pellets were quickly thawed in 37°C water bath and resuspended in swelling buffer (0.1 M Tris-HCl pH 7.0, 10 mM KOAc, 15 mM MgOAc, freshly added 1% NP-40, 1 mM DTT, 1 mM PMSF, complete protease inhibitor [Sigma] and 0.1 Unit/microliter Superase-In [Ambion]) for 10 minutes on ice. Cell suspension was then douced with a B-pestle and nuclei were pelleted at 2500× g for 5 minutes. Nuclei were lysed in nuclear lysis buffer (50 mM Tris-HCl pH 7.0, 10 mM EDTA, 1% SDS, and freshly added 1 mM DTT, 1 mM PMSF, complete protease inhibitor and 0.1 Unit/microliter Superase-In) on ice for 10 minutes, and then subjected to sonication (Misonix with a microtip) to solubilize the chromatin and shear the DNA in the size range of 250–500 bp. 100 µl of sheared chromatin was saved as “Input” DNA, it was subjected to RNase, Proteinase K, and extracted with equal volume phenol∶chloroform∶isoamyl before being precipitated with 100% ethanol. The sheared chromatin was diluted in two times hybridization buffer (750 mM NaCl, 1% SDS, 50 mM Tris-HCl pH 7.0, 1 mM EDTA, 15% formamide, freshly added 1 mM DTT, 1 mM PMSF, complete protease inhibitor and 0.1 Unit/microliter Superase-In). A total of 100 pmol of probes (either specific for PAN or LacZ) were added to 3 ml of diluted chromatin, and rotated at 37°C for 4 hours. Streptavidin-magnetic C1 beads (Invitrogen) were washed three times in nuclear lysis buffer, blocked with 500 ng/µl yeast RNA (Ambion), 100 µg/ml sheared salmon sperm DNA (Ambion) and 1 mg/ml BSA (Sigma) for 1 hour at room temperature and washed three times again in nuclear lysis buffer before resuspending in its original volume. 100 µl of blocked and washed beads were added per 100 pmol of probes, and the whole reaction mixture was rotated at 37°C for 30 minutes. Using a magnet the beads were precipitated and washed two times with 40× bead volume of 2× SSC wash buffer (2× SSC, 0.5% SDS, and freshly added 1 mM DTT, 1 mM PMSF), then two times in 1× SSC wash buffer (1× SSC, 0.5% SDS, and freshly added 1 mM DTT, 1 mM PMSF), and finally 2 times in 0.1× SSC wash buffer (0.1× SSC, 0.5% SDS, and freshly added 1 mM DTT, 1 mM PMSF). After last wash all residual liquid was removed from the beads. The DNA was eluted from the beads using 3× original volume of DNA elution buffer (50 mM NaHCO_3_, 1% SDS, 200 mM NaCl) with 100 µg/ml RNase A (Affymetrix) and 0.1 units/µl RNase H (Ambion). RNase elution was performed twice at 37°C for 20 minutes and eluent from both steps were combined. Proteinase K was added (0.2 units/µl) and incubated at 65°C for 45 minutes. DNA was extracted with equal volume phenol∶chloroform∶isoamyl (Affymetrix) and precipitated with 100% ethanol at −80°C overnight. Eluted DNA was resuspended in 50 µl of TE and used for PCR analysis. The following PCR primers were used in the PCR reactions, ORF50 promoter (Forward: GCGTGAGATGTGACCAATAGGGTG, Reverse: CGGCATATGGAGTAGTTCTAGAGTG) and K6 (Forward: CGCCTAATAGCTGCTGCTACGG, Reverse: TGCATCAGCTGCCTAACCCA).

**Table 2:** Oligonucleotides used for ChIRP.

Oligos	Sequence (5′ to 3′)	Oligos	Sequence (5′ to 3′)
PAN_1	aaaatccataggtgaagcgg	LacZ_1	ccagtgaatccgtaatcatg
PAN_2	aatgaaaaccagaagcggca	LacZ_2	tcacgacgttgtaaaacgac
PAN_3	attgccaaaagcgacgcaat	LacZ_3	attaagttgggtaacgccag
PAN_4	aagtaggacggaaaacctag	LacZ_4	aggttacgttggtgtagatg
PAN_5	cgcggtgttttttgtactac	LacZ_5	aatgtgagcgagtaacaacc
PAN_6	aatcgcagcttttgttctgc	LacZ_6	gtagccagctttcatcaaca
PAN_7	aaggggtgactgtatagttg	LacZ_7	aataattcgcgtctggcctt
PAN_8	ataaaagggggctacaactg	LacZ_8	agatgaaacgccgagttaac
PAN_9	cgctgctttcctttcacatt	LacZ_9	aattcagacggcaaacgact
PAN_10	atgagcagataggtagtgca	LacZ_10	tttctccggcgcgtaaaaat
PAN_11	ccgctttctagaattacctc	LacZ_11	atcttccagataactgccgt
PAN_12	cagattgtcacatttagggc	LacZ_12	aacgagacgtcacggaaaat
PAN_13	gcactagcctgatacaatct	LacZ_13	gctgatttgtgtagtcggtt
PAN_14	atcgacttgcttacactgga	LacZ_14	ttaaagcgagtggcaacatg
PAN_15	ttctgacaaatgccacctca	LacZ_15	aactgttacccgtaggtagt
PAN_16	cacaccactttagtccaatg	LacZ_16	ataatttcaccgccgaaagg
PAN_17	ctggaacttccaacacaaca	LacZ_17	tttcgacgttcagacgtagt
PAN_18	caacgctttcacctacaaga	LacZ_18	atagagattcgggatttcgg
PAN_19	caaggaaaaacccagccaaa	LacZ_19	accattttcaatccgcacct
PAN_20	taaaaaccttgccgtctggt	LacZ_20	ttaacgcctcgaatcagcaa

### Analysis for enrichment of PAN RNA from ChIRP

To assess the specificity of PAN RNA ChIRP chromatin and beads were prepared as described above, except that RNA was eluted from the beads in RNA pK buffer (100 mM NaCL, 10 mM Tris-HCl pH 7.0, 1 mM EDTA, 0.5% SDS) and Proteinase K was added (0.2 units/µl) and incubated at 65°C for 45 minutes followed by boiling for 15 minutes and trizol∶chloroform extraction. Input RNA and post-ChIRP hybridization supernatant was also subjected to proteinase K treatment, boiling and trizol∶chloroform extraction. Eluted RNA was treated with DNase using Turbo DNA-Free (Ambion). cDNA was generated by reverse-transcriptase PCR using iScript (BioRad). cDNA was then used for qPCR using Taqman probes specific for PAN and cyclophilin (ABi). The enrichment of PAN RNA was calculated by comparing PAN RNA eluted from the beads to PAN RNA depleted from the hybridization supernatant relative to LacZ control oligonucleotides. Both were normalized to the overall input and amount of cyclophilin (to determine non-specific RNA binding) present in each specific sample.

## Results

### Generation of recombinant KSHV BACmid with the duplicated region deleted

**Figure 1:**
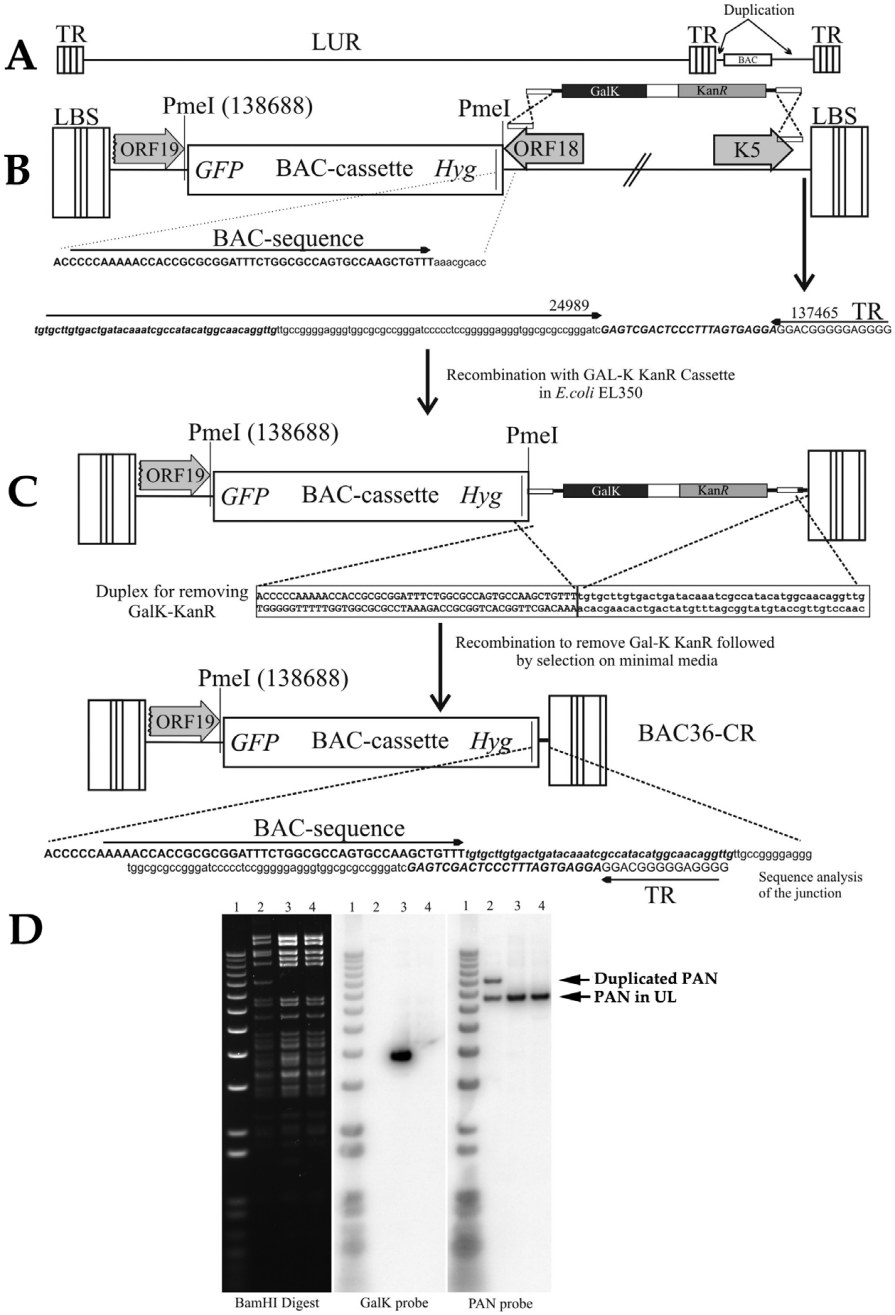
Deletion of the duplicated region within BAC36. (A) Duplicated genomic region is located between two terminal repeat sequences of the BAC36 genome (B) The duplicated ORFs 18-K5 were removed by insertion of a GalK-KanR cassette using oligonucleotides homologous to regions outside of the duplicated region. (C) Replacement of the GalK-KanR cassette in the BAC36 genome to yield the recombinant BACmid BAC36CR where the entire duplicated regions was removed. Shown is the DNA sequence after removal of the cassette (D) Ethidium bromide stained gel and Southern blot of BAC36 and BAC36CR DNA cleaved with BamHI and hybridized with either a probe specific for the GalK-KanR cassette (GalK probe) or the PAN RNA locus (PAN probe). Lanes: 1, MW marker; 2, BAC36, 3, BAC36+GalK-KanR cassette; 4, BAC36CR. Arrows indicated the PAN RNA locus in the unique long region of the genome and the duplicated PAN RNA locus located between the terminal repeats.

Our laboratory and others have used BAC36 as a template for the generation of several recombinant BACmids for mutagenesis. However the recent discovery of the duplication of a large region of the KSHV genome within this BACmid has hampered efforts to introduce mutations or deletions in genes present within the duplicated region. Specifically, ORFs 18-K5, including the PAN RNA locus, are present within the BAC36 genome twice [20]. Also, the original reported placement of the BAC cassette, containing the BACori, drug resistance genes and the EGFP ORF, was incorrect [22]. The BAC cassette in the BAC36 genome is present between two terminal repeat sequences on the right end of the genome (Fig. 1A, BAC).

In order to generate a BACmid template such that recombinants could be generated with mutations or deletions in ORFs 18-K5 including the PAN RNA locus, we decided to remove the duplicated region from the original BAC36 genome. The first step was to remove the duplicated region and replace it with a cassette that contained the gene for kanamycin resistance (KanR) and the counter-selection marker *gal K* (Fig. 1B). This recombination event removed ORFs18-K5 in the duplicated region. In order to ensure that removal of ORFs18-K5 occurred in the duplicated region and not in the unique coding region of the genome, we designed PCR primers with flanking sequence that was homologous to terminal repeat region and the 3-prime end of the hygromycin resistance gene (Fig. 1B) and performed homologous recombination in bacteria. Bacterial colonies were picked and the correct recombinants were selected and used for the second round of recombination where the GalK-KanR cassette was removed such that ORFs18-K5 duplicated region was completely eliminated (Fig. 1C). The result of this final recombination event was the generation of BAC36CR (Fig. 1C). A Southern blot of the BAC36CR recombinant shows that the duplicated region of BAC36 was removed by the first round of recombination (Fig. 1D, compare lanes 2 and 3, PAN probe). All recombinants were confirmed by DNA sequencing.

**Figure 2:**
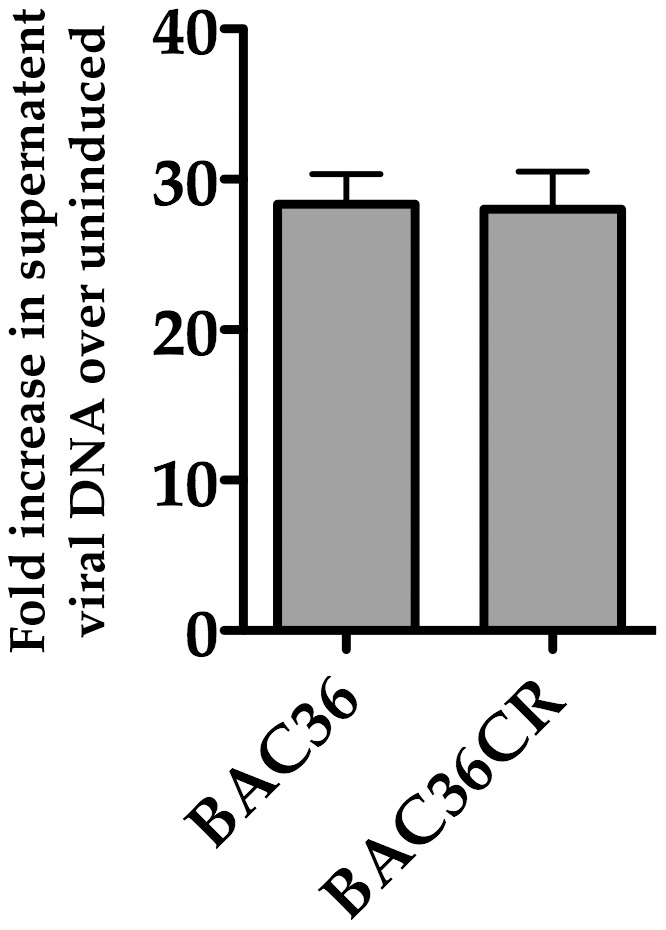
Production of infectious virus from BAC36CR induced cell lines. BAC36 and BAC36CR cell lines were induced with TPA/n-butyrate for 5 days and supernatant virus was analyzed using qPCR. The amount of virus DNA accumulation was compared to DNA accumulation from uninduced BAC36. The error bars are the standard deviation of the mean from 3 separate experiments.

We next transfected BAC36CR into 293L cells and selected colonies using hygromycin. A selected colony was expanded and treated with TPA/n-butyrate for 5 days and supernatant virus was collected and virus DNA was measured using qPCR. Virus DNA collected from BAC36CR infected cell supernatants was subjected to qPCR and compared to virus DNA obtained from similarly treated original BAC36 containing cell lines. Supernatant virus DNA was compared to BAC36CR untreated cells supernatants. Similar levels of virus DNA accumulated from cells containing BAC36 and BAC36CR (Fig. 2). These data showed that we generated a BACmid clone, based on the original BAC36 template, which did not contain the duplicated ORFs and only one PAN RNA locus and replicated to the same efficiency as the original BAC36 clone.

### Generation of a recombinant BACmid with the PAN RNA locus deleted

**Figure 3:**
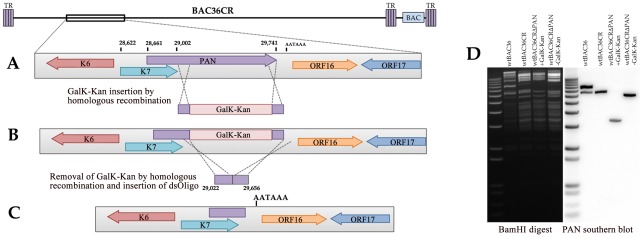
Generation of a recombinant BACmid with the PAN RNA locus deleted, BAC36CRΔPAN. (A) The BAC36CR template was used to insert the GalK-KanR cassette such that 634 nts of the PAN RNA gene was removed from the genome. (B) The GalK-KanR cassette was removed by homologous recombination and reverse selection. (C) BAC36CRΔPAN was generated by removal of the GalK-KanR cassette and the putative polyadenlyation signal downstream of the original PAN RNA and K7 genes was preserved. (D) Ethidium bromide stained agarose gel and Southern blot of BAC36, BAC36CR and BAC36CRΔPAN DNA cleaved with BamHI showing the removal of part of the PAN RNA locus.

The generation of BAC36CR now allowed for the production of recombinant BACmids with mutations or deletions in regions of the KSHV genome that encoded ORFs K18-K5. Since our laboratory is interested in the function and contribution of PAN RNA to virus growth we decided to delete the PAN RNA locus from BAC36CR. The KSHV PAN RNA locus is located between nucleotides 28661–29741; a portion of the K7 ORF overlaps with the PAN transcription unit (Fig. 3A). Hence, any plan involving the deletion of the PAN RNA locus would have to preserve the K7 ORF along with the putative polyadenlyation signal down stream of PAN RNA (Fig. 3A). Again using the insertion of the GalK-KanR cassette we removed nucleotide sequences from 29022–29656 (Fig. 3B). This deletion removed 634 nts of the PAN RNA transcript (Fig. 3B). Subsequent removal of the GalK-KanR cassette resulted in the deletion of a large region of the PAN RNA transcript, preservation of the K7 ORF and placement of the putative polyadenylation site just downstream of the K7 ORF (Fig. 3C). BAC36, BAC36CR and BAC36CRΔPAN DNA was cleaved with BamHI and separated by agarose gel electrophoresis and transferred to a nylon membrane. BACmid DNA was hybridized with a radiolabeled probe complementary to the PAN RNA locus. Cleavage with BamHI allows for the visualization of the duplicated region in the original BAC36, which is shown by the presence of two distinct bands when hybridized to a PAN probe (Fig. 3D, BAC36 lane). Also, shown is the BAC36CR DNA BamHI cleavage where only one band is present corresponding to the single PAN RNA locus (Fig. 3D, BAC36CR lane). The BAC36CRΔPAN BamHI cleaved DNA shows the presence of a smaller band confirming the deletion of 634 nts from the PAN RNA locus (Fig. 3D, lane BAC36CRΔPAN). The deletion was subsequently confirmed by DNA sequence analysis.

### PAN RNA expression is required for production of supernatant virus

**Figure 4:**
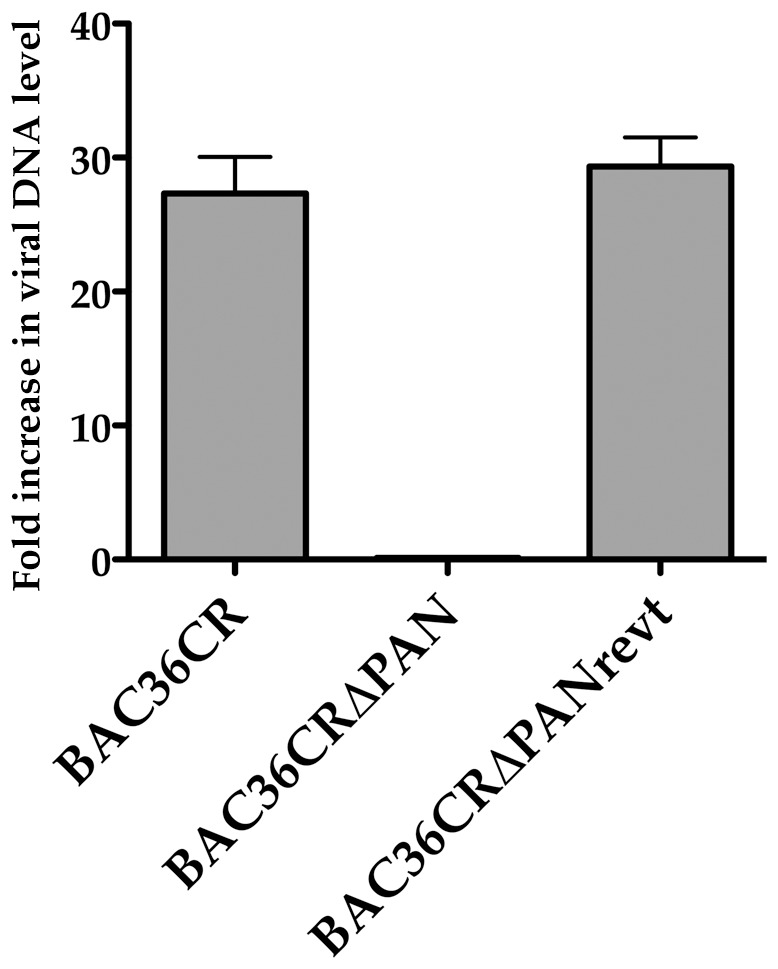
BAC36CRΔPAN fails to produce supernatant virus. Supernatant virus was harvested from cell lines harboring BAC36CR, BAC36CRΔPAN or BAC36CRΔPANrevt DNA 5 days post induction. Relative amounts of viral DNA was analyzed using qPCR and is reported as fold increase in viral DNA level compared to uninduced BAC36CR.

Once a PAN RNA deleted virus was generated the next step was to evaluate the growth characteristics of a virus that failed to express PAN RNA. To this end, BAC36CRΔPAN DNA was transfected into 293L cells and individual colonies were selected. Colonies harboring BAC36CRΔPAN, BAC36CR and revertant BACmid BAC36CRΔPANrevt were expanded and treated with TPA/n-butyrate for 5 days. Total cellular RNA and cell supernatants were collected. We measured supernatant virus accumulation by qPCR compared to uninduced BAC36CR collected samples. No virus DNA was detected in the BAC36CRΔPAN supernatant samples (Fig. 4). BAC36 and the revertant BACmid showed an increase in supernatant virus upon induction (Fig. 4). Since this observation could be due to a significant difference in the number of latent viral genomes between the wt and mutant viruses, we also measured the number of viral genomes present in wt BAC36CR and BAC36CRΔPAN. qPCR analysis showed that viral copy numbers were similar between the two BAC containing cells lines (∼20 copies per cell). The lack of production of supernatant virus for the PAN RNA deletion mutant suggests that expression of the transcript is essential for virus growth.

### The lack of PAN RNA expression results in a decrease in viral gene expression

Since we did not observed the production of supernatant virus from induced cells harboring BAC36CRΔPAN, we next investigated mRNA accumulation levels for specific viral genes. BAC36CRΔPAN and BAC36CR containing cell lines were treated with TPA/n-butyrate and total cellular RNA was harvested at 4 days post induction. We measured mRNA accumulation for the viral encoded mRNAs: ORF50, PAN, K7, K-bZIP (K8), ORF25, ORF57 and LANA (ORF73).

**Figure 5:**
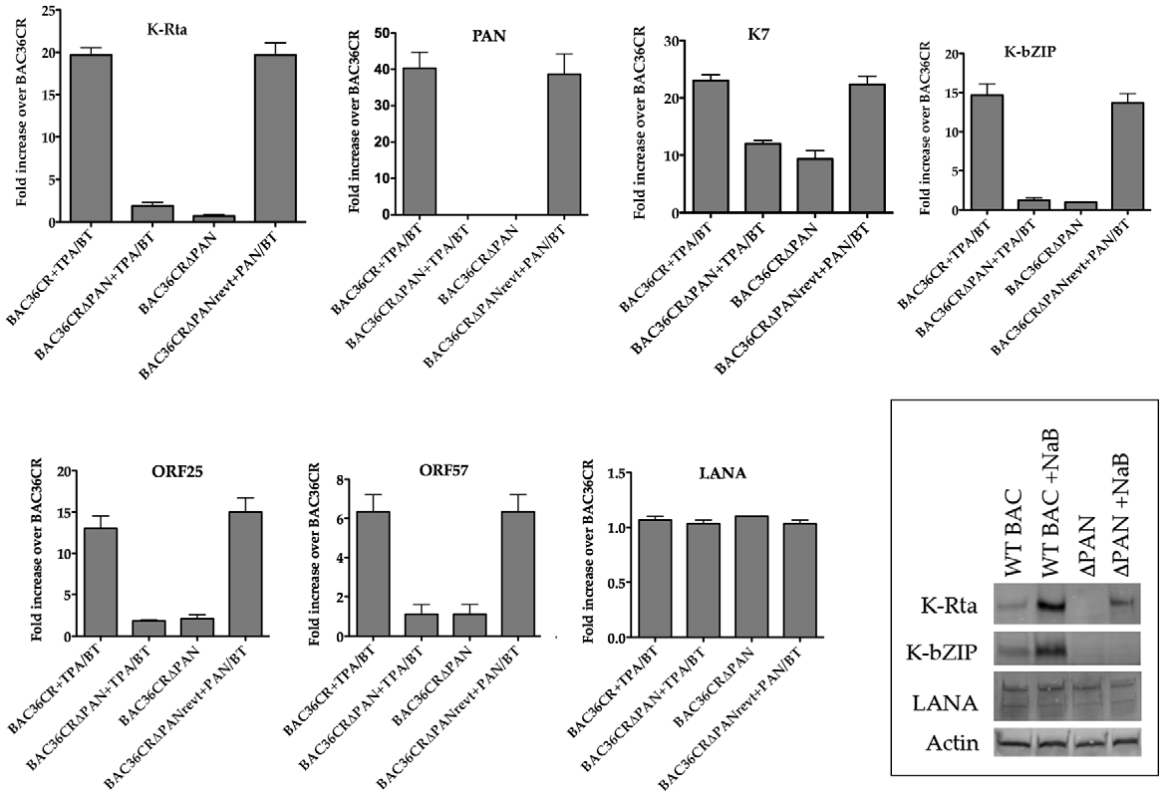
Decreased gene expression in the absence of PAN RNA expression. qPCR analysis of mRNA accumulation from genes encoding K-Rta, K7, K-bZIP, ORF25 and ORF57 from BAC36CR, BAC36CRΔPAN or BAC36CRΔPANrevt cells treated with TPA/n-butyrate for 4 days. Values are compared to mRNA accumulation from uninduced BAC36CR containing cells. Each experiment was performed 3 times and error bars are the standard deviation from the mean. **Boxed Panel.** Western blot analysis of protein extracts reacted with anti-K-Rta, anti-K-bZIP, anti-LANA or anti-actin specific antibodies from induced and uninduced cell lines containing BAC36CR or BAC36CRΔPAN.

Interestingly, the mRNA accumulation level for the major transactivator K-Rta (ORF50) was about 10-fold less in BAC36CRΔPAN containing cells lines (Fig. 5). This suggested that the absence of PAN RNA had a broad impact of virus gene expression. The expression of PAN RNA, as expected, was not detected in BAC36CRΔPAN cells (Fig. 5). The expression of K7, a gene that partially overlaps with the PAN RNA gene, was detected with mRNA accumulation levels about 10-fold less than those observed in cells containing BAC36CR (Fig. 5). The detection of K7 expression indicated that the deletion of the PAN RNA locus and the placement of the putative polyadenlyation signal just downstream of the K7 ORF allowed for efficient expression of the K7 transcript. As expected the expression level of the early gene K-bZIP (K8) was reduced in induced cells harboring BAC36CRΔPAN compared to BAC36CR or revertant virus (Fig. 5). The accumulation of the major capsid protein transcript encoded by ORF25 was also reduced approximately 10-fold compared to the level observed from BAC36CR containing cells (Fig. 5). We also investigated the mRNA accumulation level for ORF57 transcripts. Again, the level of ORF57 transcripts in BAC36CRΔPAN containing cells lines was reduced when compared to mRNA levels seen in the wild type BACmid BAC36CR (Fig. 5). The level of LANA mRNA was not affected by the lack of PAN RNA expression, suggesting that PAN RNA did not impair the establishment or maintenance of latent KSHV genomes (Fig. 5).

We also evaluated protein accumulation for K-Rta, K-bZIP and LANA in induced and uninduced cells harboring BAC36CRΔPAN or BAC36CR by Western blot. Protein lysates were prepared from cells harboring BAC36CRΔPAN or BAC36CR either induced with TPA/Butyrate or uninduced. Lysates were resolved by SDS PAGE and separated proteins were transferred to PVDF membranes and reacted with antibodies specific for K-Rta, K-bZIP or LANA proteins.

Consistent with qPCR results the level of K-Rta protein was reduced upon induction in cells containing BAC36CRΔPAN compared to BAC36CR (Fig. 5, Boxed panel). K-Rta was not detected in protein lysates from uninduced BAC36CRΔPAN containing cells, whereas a typical basal level of K-Rta (due to spontaneous lytic reactivation) was observed in protein lysates from wild type BAC36CR cell lines (Fig. 5, Boxed panel). The level of K-bZIP protein was undetectable in protein lysates generated from BAC36CRΔPAN, consistent with the overall observed decrease in gene expression when PAN RNA is not expressed (Fig. 5, Boxed panel). The protein levels of LANA was similar in BAC36CRΔPAN and BAC36CR cell lines again suggesting that LANA in unaffected by PAN RNA (Fig. 5, Boxed panel). As a control for protein loading we reacted blots with anti-actin antibody (Fig. 5, Boxed panel).

These results show that PAN RNA contributes to overall gene expression and suggests a role for the transcript in mediating the enhanced expression of the major transactivator K-Rta.

### K-Rta expression is decreased at early times post induction

**Figure 6:**
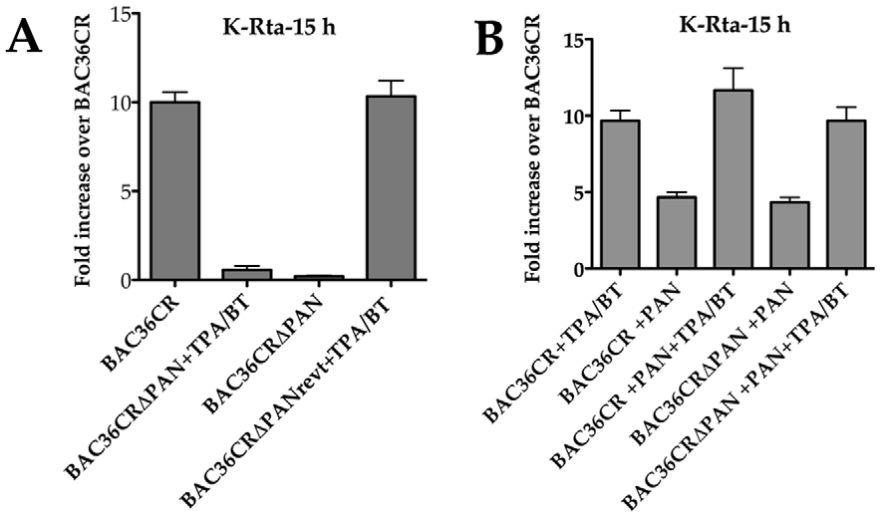
K-Rta mRNA accumulation is decreased at early times post induction in the absence of PAN RNA expression. (A) qPCR analysis of K-Rta mRNA accumulation from BAC36CR or BAC36CRΔPAN containing cells treated with TPA/n-butyrate (TPA/BT) for 15 hr. The experiment was performed 3 times and error bars are the standard deviation from the mean. (B) **Expression of PAN RNA in** ***trans*** **activates K-Rta transcription BACmid harboring cell lines.** qPCR analysis of K-Rta mRNA accumulation from BAC36CR or BAC36CRΔPAN containing cells transfected with a PAN RNA expression plasmid with or without treatment with TPA/n-butyrate (TPA/BT). Total RNA was harvested 15 h post induction. The experiment was performed 3 times and error bars are the standard deviation from the mean.

Our analysis of K-Rta gene expression was performed at 4 days post induction. To evaluate if the mRNA accumulation of K-Rta is affected by the absence of PAN RNA at early times post induction, we performed the same experiment using BAC36CRΔPAN and BAC36CR cell lines induced with TPA/n-butyrate except we evaluated K-Rta expression at 15 h post induction. K-Rta transcript levels were also reduced at this early time point (Fig. 6A) suggesting that PAN RNA is required for efficient initial transactivation of K-Rta.

Since K-Rta expression was apparently decreased in cell containing BAC36CRΔPAN, we evaluated the ability of PAN RNA to transactivate K-Rta expression. BAC36CRΔPAN or BAC36CR cell lines were transfected with a PAN RNA expression plasmid and treated with TPA/n-butyrate (TPA/BT). At 15 h post induction total cellular RNA was harvested and the level of K-Rta mRNA accumulation was measured using qPCR. In all cases, induced or uninduced, the expression of PAN RNA did result in an increase in K-Rta expression from BACmid DNA (Fig. 6B). These results suggested that PAN RNA could transactivate expression of K-Rta.

### Expression of K-Rta in *trans* does not complement BAC36CRΔPAN

**Figure 7:**
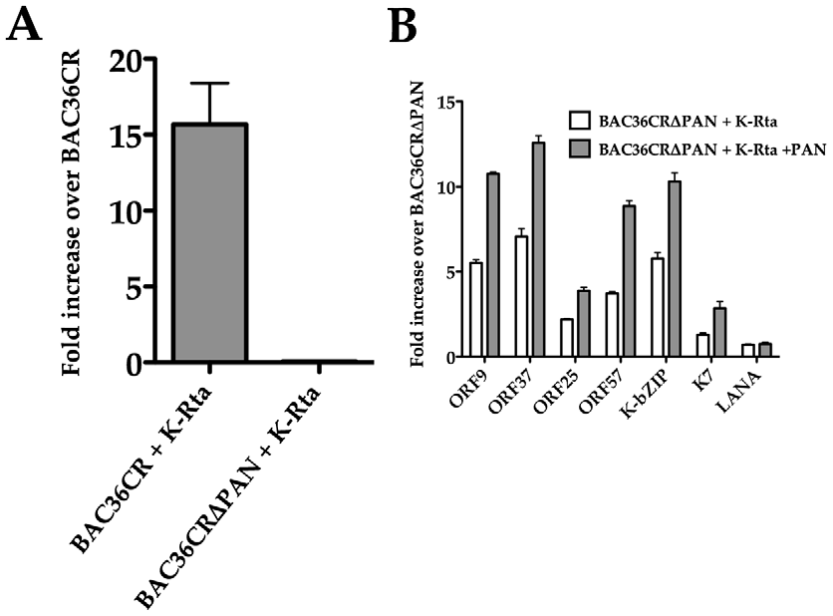
Overexpression of K-Rta cannot complement BAC36CRΔPAN. (A) BACmid containing cell lines were transfected with a K-Rta expression plasmid and supernatant virus DNA was measured 4 days post transfection. The experiment was repeated 3 times. Error bars are the standard deviation from the mean. (B) *Trans* expression of K-Rta activates viral promoters in BAC36CRΔPAN containing cells and expression is enhanced in the presence of PAN RNA. BAC36CRΔPAN containing cells were transfected with K-Rta with or without the cotransfection of the PAN RNA expression plasmid and qPCR analysis was performed to measure mRNA accumulation for several viral encoded genes.

The suppression of K-Rta expression in absence of PAN RNA suggested that the PAN transcript provides an essential function for the expression of K-Rta. Therefore the lack of virus production observed from induced BAC36CRΔPAN harboring cells could be due to the general lack of K-Rta expression, which would result in an overall lack of gene expression and a subsequent lack of virus production. Hence, we investigated the possibility if the expression of K-Rta in *trans* could complement BAC36CRΔPAN and lead to the production of supernatant virus. This would mean that the observed defect in BAC36CRΔPAN is due to the lack of adequate K-Rta expression, which could be overcome by the expression of K-Rta in *trans*. To this end we transfected BAC36CRΔPAN and BAC36CR cell lines with a plasmid that expresses K-Rta. Cell supernatants were collected at 4 days post transfection and the amount of virus DNA was measured by qPCR. No virus DNA was detected in the supernatant from cells containing BAC36CRΔPAN transfected with the K-Rta expression plasmid (Fig. 7A). Control samples from the BAC36CR cell line transfected with the K-Rta expression plasmid did produce supernatant viral DNA (Fig. 7A). This result strongly suggested that PAN RNA supplies an essential function for virus replication in addition to the transactivation of K-Rta.

Since we did not observe virus production from cells containing BAC36CRΔPAN transfected with a K-Rta expression plasmid, we investigated if viral gene expression could occur in cells harboring BAC36CRΔPAN in the presence of K-Rta expressed in *trans*. We also wanted to investigate if the addition of PAN RNA has an effect on gene expression in the presence of K-Rta *trans* expression. BAC36CRΔPAN containing cells were transfected with the K-Rta expression plasmid with or without the addition of the PAN RNA expression plasmid. qPCR analysis was performed for each transfection. An increase in viral mRNA accumulation was observed when K-Rta was supplied in *trans* (Fig. 7B). The presence of both K-Rta and PAN RNA expression plasmids had an enhanced effect on gene expression (Fig. 7B). These data suggested that expression of K-Rta was capable of upregulating certain promoters in the absence of PAN RNA expression and that PAN RNA could enhance expression from the KSHV genome. This result also confirmed that PAN RNA supplied an additional required function or perhaps was facilitating viral gene the expression from the ORF50 promoter and/or other viral promoters.

### PAN RNA physically interacts with the K-Rta promoter

The activity of lncRNAs usually involves a mechanism where the transcript interacts with chromatin and mediates changes in chromatin structure. Since the studies involving BAC36CRΔPAN demonstrated that PAN RNA potentially regulates the expression of K-Rta, we next investigated if PAN RNA interacts with the K-Rta promoter region. An interaction of PAN RNA with the ORF50 promoter would be consistent with a model that suggests the targeting of chromatin modifying proteins to key regions controlling gene expression. In this specific case, PAN RNA would mediate the transcriptional activation or enhancement of K-Rta expression. To examine if PAN RNA does indeed interact with the K-Rta promoter we adapted a method called “Chromatin isolation by RNA purification” (ChIRP) for PAN RNA. With this powerful method, it is possible to identify the target DNA sequence that interacts with a specific lncRNA [21]. To identify an interaction between PAN RNA and the K-Rta promoter we used the ChIRP protocol followed by a PCR amplification of the K-Rta promoter region. Therefore, if PAN RNA interacts with the K-Rta promoter region of the KSHV genome we would be able to detect this interaction using specific PCR primers.

TREx/BCBL-1 Rta cells were treated with doxycycline (DOX) to induce lytic replication. Cells were incubated for 3 days followed by isolation of DNA occupied by PAN RNA using the ChIRP method. Using a series of generated biotinylated oligonucleotides that hybridize to PAN RNA, we performed the ChIRP assay where the end product was DNA that was occupied by PAN RNA. We then used the isolated DNA as a substrate for PCR amplification using primers specific for the K-Rta promoter or control K6 ORF coding sequence. As a control for nonspecific binding, we used biotinylated oligonucleotides for the unrelated LacZ RNA for pull downs in TREx/BCBL-1 Rta cells.

**Figure 8:**
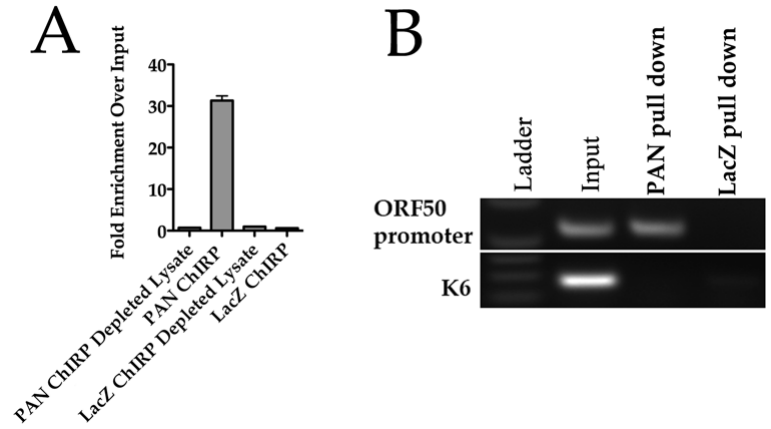
PAN RNA physically interacts with the ORF50 promoter. (A) PAN RNA is enriched 30-fold by ChIRP assay. PAN RNA or LacZ specific biotinylated oligonucleotides were used to enrich PAN RNA. Recovered RNA or RNA from the depleted lysate (post ChIRP) was measured by qPCR. (B) TREx/BCBL-1 Rta cells were treated with DOX and 3 days post treatment ChIRP assays were performed. Tiling biotinylated oligonucleotides were used that hybridized to either PAN RNA (20 oligonucleotides) or control LacZ RNA (20 oligonucleotides). Pulled down DNA that was occupied by RNA was amplified using primers specific for the ORF50 promoter region or K6 ORF coding sequence.

**Figure 9:**
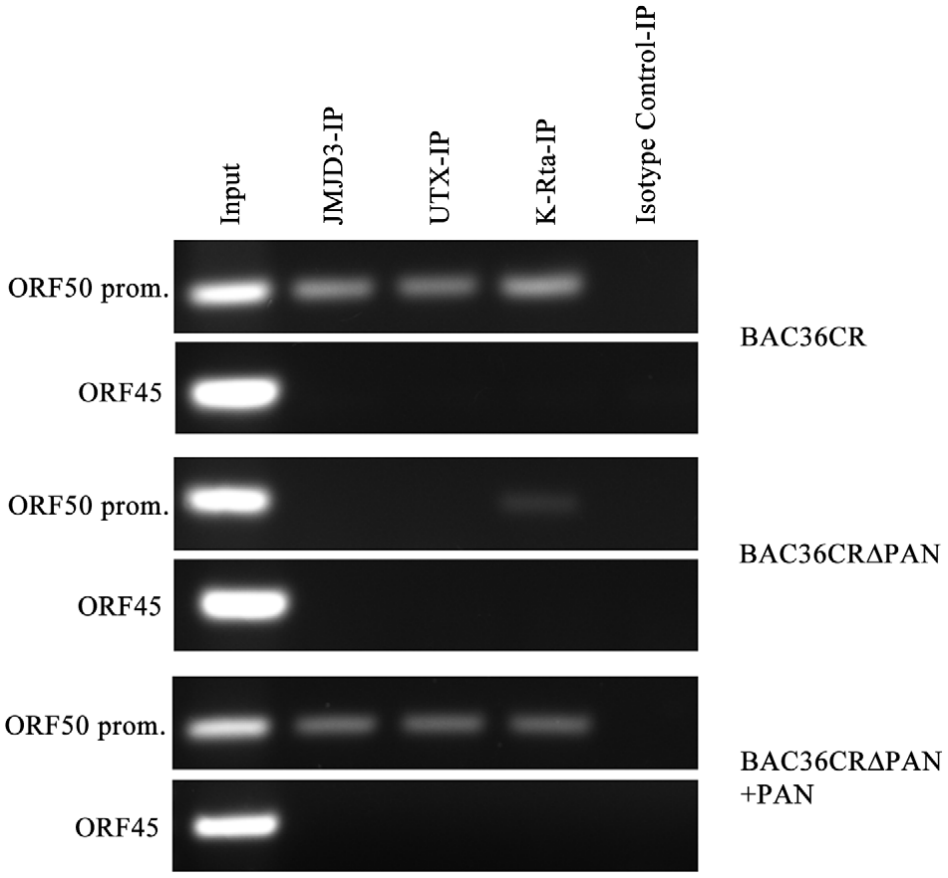
JMJD3 and UTX demethylases interact with KSHV DNA in the presence of PAN RNA expression. Cell lines containing BAC36CR or BAC36CRΔPAN were transfected with a K-Rta expression plasmid and ChIP assays were performed 3 days post transfection. Immunoprecipitations were performed using antibodies specific for JMJD3, UTX, K-Rta or an isotype specific antibody control. PCR primers specific for the ORF50 promoter or ORF45 were used to amplify immunoprecipitated DNA. Panel BAC36CRΔPAN+PAN: cells were transfected with both a K-Rta and PAN RNA expression plasmid.

In order to determine the efficiency of our ChIRP protocol for specifically enriching PAN RNA, we measured the amount of PAN transcript that was recovered from the ChIRP pull down by qPCR. The relative fold enrichment over input was determined for both PAN RNA specific oligonucleotides and control LacZ oligonucleotides (Fig. 8A). We also evaluated the amount of PAN RNA that remained in the depleted lysate after the oligonucleotide-streptavidin pull down (Fig. 8A). PAN RNA was enriched approximately 30-fold in the PAN specific oligonucleotide pull downs (Fig. 8A, PAN ChIRP), whereas very small amounts of PAN RNA was detected in the LacZ oligonucleotide specific ChIRP pull downs (Fig. 8A, LacZ ChIRP). Also, we observed an almost compete recovery (98%) of PAN RNA from input samples indicating that our ChIRP protocol was extremely efficient for enrichment of PAN RNA (Fig. 9A, PAN ChIRP Depleted Lysate).

Hence once we determined that our PAN RNA ChIRP assay was an efficient means to recover PAN RNA from lytically induced cells, we then examined the ability of PAN RNA to interact with the ORF50 promoter region. ChIRP pull downs were treated as described in materials and methods and the resulting DNA product was subjected to PCR amplification using primers specific for the ORF50 promoter region. A specific PCR product was detected in the sample using PAN RNA specific oligonucleotides whereas no specific amplification product was observed in control pull downs or amplification of the K6 promoter region (Fig. 8B). This result demonstrates that PAN RNA interacts with the ORF50 promoter and suggests that activation of transcription could involve a mechanism where PAN RNA facilities the remodeling of KSHV chromatin.

### Demethylases JMJD3 and UTX interact with the ORF50 promoter

Previous data showed that most of the KSHV latent genome is repressed by the presence of the H3K27me3 mark [23]. Further, it was demonstrated that when the lytic cycle is induced, KSHV genomes show an increase in activation histone marks such as H3K4me3 and acetylated histones and a decrease in the repressive H3K27me3 mark [23]. One mechanism for the removal of the repressive H3K4me3 mark is the targeting of specific demethylases for this repressive mark. The demethylases JMJD3 and UTX were shown to specifically remove polycomb group proteins marking on chromatin [24]–[26]. Evidence suggests that the transfection of a plasmid expressing demethylases is sufficient to activate lytic replication and hence the removal of the specific repressive H3K27me3 mark is important for lytic reactivation of latent KSHV genomes [23]. Hence we investigated the possibly that JMJD3 and UTX interact with the ORF50 promoter in the presence of PAN RNA expression. We also evaluated the ability of K-Rta to interact with the ORF50 promoter. Since it was previously shown that K-Rta interacts with its own promoter [27]–[29], we were interested if the expression of PAN RNA affected the ability of K-Rta to bind to the ORF50 promoter.

BAC36CR or BAC36CRΔPAN containing cell lines were induced by transfection of a plasmid that expressed K-Rta. As shown previously, the transfection of K-Rta is insufficient to reactivate BAC36CRΔPAN. Three days post transfection ChIP assays were performed on transfected BACmid containing cell lines using antibodies specific for JMJD3, UTX, K-Rta or an isotype control. Immunoprecipitated DNA was amplified using PCR primers specific for the ORF50 promoter or the K6 gene coding sequence. In induced BAC36CR containing cells, JMJD3 and UTX interacted with the ORF50 promoter region (Fig. 9, BAC36CR, ORF50 promoter, Panel: BAC36CR). Whereas in K-Rta transfected BAC36CRΔPAN containing cell lines no interaction was detected (Fig. 9, BAC36CRΔPAN, ORF50 promoter, Panel: BAC36CRΔPAN). K-Rta is known to bind to and transactivate it's own promoter, in our study only a slight interaction of K-Rta with the ORF50 promoter was detected in the BAC36CRΔPAN cell line (Fig. 9, K-Rta-IP, ORF50 promoter, Panel: BAC36CRΔPAN).

To confirm that the interaction of JMJD3, UTX and K-Rta is mediated by the presence of PAN RNA, we next cotransfected a PAN RNA expression plasmid along with the K-Rta expression plasmid into cells containing BAC36CRΔPAN (Fig. 9, Panel: BAC36CRΔPAN+PAN). If PAN RNA is required for the interaction of the demethylases and K-Rta with the ORF50 promoter then we would expect that ChIP analysis would show these proteins bound to KSHV genomic DNA. ChIP assays confirm that, JMJD3, UTX and K-Rta were all shown to interact with the ORF50 promoter when PAN RNA was supplied in *trans*. Hence the presence of PAN RNA is required for the interaction of these proteins with the KSHV genome.

These data suggests that PAN RNA expression may facilitate and enhance the interaction of K-Rta with target DNA and could be activating transcription by facilitating the targeting of demethylases to the ORF50 promoter.

### JMJD3, UTX and MLL2 interact with PAN RNA

Since we show that PAN RNA interacts with and up regulates the K-Rta promoter and we detected the presence of JMJD3 and UTX at the ORF50 promoter, we investigated the possibility that PAN RNA interacts with these specific demethylases. The observation that JMJD3 and UTX interact with the K-Rta promoter suggests that the removal of the repressive histone mark is a possible mechanism for activation of transcription. Therefore if PAN RNA interacts with UTX and/or JMJD3 then this would strongly suggests that PAN RNA targets these specific demethylases to KSHV promoters to activate transcription. Also, it is known that UTX interacts with MLL2 (a thorax-group protein) [24]. MML2 is a histone-lysine N-methyltransferase, which methylates H3 at the K4 position (activation mark). Therefore we also investigated if PAN RNA interacts with MML2.

**Figure 10:**
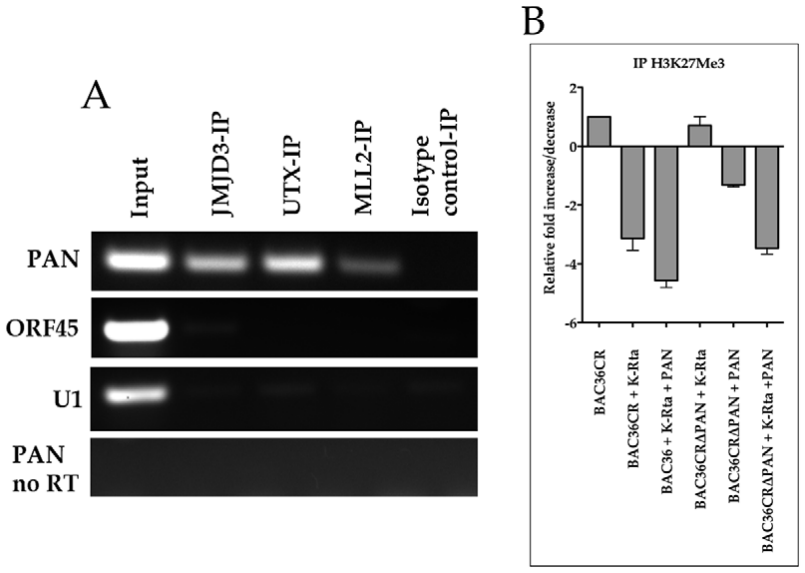
PAN RNA interacts with demethylases and the histone methyltransferase MLL2. (A) TREx/BCBL-1 Rta cells were treated with DOX and RNA CLIP assays were performed 3 days post treatment. PAN RNA-protein complexes were immunoprecipitated using anti-JMJD3, anti-UTX, anti-MML2 or isotype control antibodies. PCR primers were used to amplify (after RT) PAN RNA, ORF45 RNA or U1 RNA. Also shown is PCR amplification without a reverse transcriptase reaction (PAN no RT). (B) PAN RNA expression leads to a relative decrease in the H3K27me3 mark on the ORF50 promoter. BAC36CR or BAC36CRΔPAN containing cells were transfected with either a K-Rta expression plasmid and/or a plasmid expressing PAN RNA. ChIP assays were performed using anti-H3K27me3 specific antibody. Immunoprecipitated DNA was analyzed by qPCR normalized to input DNA. Data is reported as fold decrease compared to BAC36CR untreated samples. Error bars are the standard deviation of the mean from three separate experiments.

We induced K-Rta expression in TREx/BCBL-1 Rta cells with treatment of DOX and performed an RNA CLIP using antibodies specific for JMJD3, UTX or MLL2. Immunoprecipitated product was reverse transcribed using Qiagen One Step and cDNA was amplified using PAN RNA specific PCR primers. For controls we immunoprecipitated with an isotype control antibody. Additionally, we used PCR primers specific for the unrelated mRNAs encoding KSHV ORF45 and cellular U1. Specific PCR amplification products were detected for PAN RNA when using specific UTX, JMJD3 and MLL2 specific antibodies (Fig. 10A, lanes JMJD3, UTX and MLL2) whereas no product was detected in control immunoprecipitations or amplifications using primers for unrelated RNAs (Fig. 10A, ORF45 and U1). These data indicate that PAN RNA interacts with the demethylases UTX and JMJD3 and with the MLL2 methyltransferase. Hence these results strongly suggest that one mechanism of gene activation is by the specific targeting of PAN RNA to KSHV promoters that are repressed with the H3K27Me^3^ mark by the PRC2. We speculate that PAN RNA mediates the removal of the repressive mark while simultaneously facilitating the activation of gene expression by the activity of MLL2.

### PAN RNA expression decreases the relative amount of H3K27me3 mark at the ORF50 promoter

Since we show that PAN RNA interacts with the demethylases UTX and JMJD3 we evaluated the amount of H3K27me3 at the ORF50 promoter in the context of the PAN RNA deletion mutant BAC36CRΔPAN. BACmid containing cell lines were transfected with either a K-Rta expression plasmid and/or a plasmid that expresses PAN RNA. The transfection of a K-Rta expression plasmid into BAC36CR containing cells resulted in the expected decrease in the H3K27me3 mark (Fig. 10B, BAC36CR+K-Rta). The cotransfection of the PAN RNA expression plasmid along with the K-Rta expression plasmid yielded an additional decrease in the H3K27Me^3^ mark (Fig. 10B, BAC36CR+K-Rta+PAN). However, in the absence of PAN RNA expression the transfection of the K-Rta expression plasmid did not significantly affect the relative level of H3K27Me^3^ at the ORF50 promoter (Fig. 10B, BAC36CRΔPAN+K-Rta). The transfection of the PAN RNA expression plasmid alone in BAC36CRΔPAN BACmid harboring cells did show a reduction of H3K27me3 at the ORF50 promoter (Fig. 10B, BAC36CRΔPAN+PAN), whereas the cotransfection of both the K-Rta and PAN RNA expression plasmids resulted in a comparable reduction of the H3K27me3 mark to that observed in BAC36CR similarly treated cells (Fig. 10B BAC36CRΔPAN+K-Rta+PAN). These data strongly suggest that PAN RNA expression leads to the activation of KSHV gene expression by facilitating changes in histone modifications by bridging and interaction of chromatin modifying enzymes with the KSHV genome.

## Discussion

The role of PAN RNA in KSHV replication and growth has been elusive partly due to the lack of a recombinant virus with a deletion in the PAN locus. Although our laboratory has generated several BACmid mutants, the generation of a PAN RNA gene deletion appeared to be difficult. The reason for the difficulty, with respect to introducing mutations or deletions within this region of the BAC36 genome, was illuminated by the recent publication demonstrating that ORFs 18, 17, 16, K7, K6, and K5, including the PAN RNA locus was duplicated in BAC36 [20]. Hence it became clear that this duplication had to be removed from BAC36 such that deletions or mutations within the PAN RNA locus could be introduced. To this end, we generated a new BACmid using the BAC36 template that removed the duplicated region. This new BACmid, referred to as BAC36CR, removed the duplicated ORFs such that only the cassette containing the BAC ori, hygromycin and chlorophenicol resistance genes and EGFP remained, flanked by the terminal repeat region. BAC36CR replicated with the same efficiency as the original BAC36. Using BAC36CR we were able to efficiently remove most of the PAN RNA gene to create BAC36CRΔPAN. Because of the complicated transcription unit for PAN RNA in that the K7 ORF partially overlaps with the PAN RNA gene, we strategically repositioned the putative K7 polyadenylation signal such that it was just downstream of the K7 ORF. Previous data suggested that the K7 transcript utilizes the polyadenylation signal downstream of the PAN RNA gene [30]. Our qPCR data indicates that the K7 transcript is produced and hence the generation of BAC36CRΔPAN strongly suggests that deletion we engineered into the genome only affects the expression of PAN RNA. Additionally, the generation of a revertant BAC36CRΔPAN BACmid clone also confirms that the observed phenotype is due to the lack of expression of PAN RNA.

Recently, there were two reports describing a role for PAN RNA in both KSHV growth and a wider role, which implicated PAN RNA in mediating the control of cellular gene expression [15], [16]. Our data from the current study is consistent with the observed defect in late gene expression and virus production observed from the knockdown of PAN RNA using antisense oligonucleotides [16]. However, antisense oligonucleotide treatment showed only a small decrease in the expression of immediate early and early genes, whereas our study shows that the absence of PAN RNA expression results in a dramatic repression of both immediate early (K-Rta and ORF57) as well as early and late gene expression. These differences are most likely due to the fact that in our system PAN RNA is not expressed whereas treatment with antisense oligonucleotides is associated with incomplete knockdown and toxicity. BAC36CRΔPAN containing cells accumulated dramatically less K-Rta mRNA suggesting that PAN RNA may be part of a feedback loop where adequate expression is required for sustained K-Rta transcription. Another possibility is that PAN RNA contributes to genome replication and subsequent accumulation of K-Rta. However, K-Rta mRNA accumulation was also depressed at early times post induction (15 h), suggesting that PAN RNA may be necessary for initial as well as sustained expression. Data suggests that PAN RNA expression is required for the efficient interaction of K-Rta with the ORF50 promoter implicating PAN RNA in facilitating protein-DNA interactions.

The observation that PAN RNA interacts with specific demethylases coupled with the data showing a physical interaction with the ORF50 promoter strongly suggests that PAN RNA acts to remove the repressive H3K27me3 mark. Also, since we detected an interaction of PAN RNA with MLL2, the activation of transcription could be due to the simultaneous marking with H3K4me3. The data presented here demonstrates that PAN RNA can mediate changes in the repressive H3K27me3 mark on KSHV chromatin. This is the first reporting of a lncRNA interacting with specific demethylases to remove the markings mediated by polycomb repressive complex (PRC) proteins. Interestingly, it was shown previously that both H3K4me3 and H3K27me3 marked the ORF50 locus during latency, with an increase in activation and decrease in repressive marks upon lytic reactivation [23]. PAN RNA could serve as the conduit for changes in histone markings across the KSHV genome. This model is consistent with the observations that a small amount of K-Rta expression was detected in the PAN RNA deletion mutant and we speculate that this interaction was insufficient to maintain K-Rta expression and fully reactivate latent genomes. However, the transfection of a PAN RNA expression plasmid was able to activate the ORF50 promoter suggesting that PAN RNA targets at least this one locus within the KSVH genome. Studies are underway to evaluate if PAN RNA interacts with other regions of the KSHV genome. Hence, the data presented here indicates that the PAN transcript acts as a molecular scaffold for chromatin modifying complexes and subsequently regulates viral gene expression. We acknowledge that the interaction of PAN RNA with demethylases JMJD3 and UTX could be indirect. It is also clear that PAN RNA has the capacity to activate or repress gene expression given the fact that we previously demonstrated repression of several cellular encoded genes [15]. As far as the repressive nature of PAN RNA, we did detect an interaction with polycomb group proteins (data not shown). Therefore PAN RNA is multifunction with respect to the regulation of gene expression. Although the mechanism proposed here involves the interaction of PAN RNA with chromatin modifying enzymes, PAN RNA was shown to interact with histones H1 and H2A [15]. Hence, these interactions allows for a mechanism in which PAN RNA directly alters the association of histones with chromatin. The interaction of histone proteins by PAN RNA could account for the general decrease in viral transcription that was observed in the absence of PAN RNA, chromatin remains in a less open configuration. Studies are underway to evaluate the total impact of PAN RNA on the KSHV genome.

Many long noncoding RNAs (lncRNA) were shown to mediate suppression or activation of gene expression [31]–[39]. PAN RNA may play a critical role in both immune suppression as well as genome replication. It is clear that lncRNAs, like PAN RNA, are multifunctional and act in a global manner through various mechanisms. The data presented here clearly shows that PAN RNA is an essential factor for virus growth and is a multifunctional regulatory transcript.
